# Weight Loss Effects of Glucagon-Like Peptide-One Receptor Analog Treatment in a Severely Obese Patient During Hospital Admission

**DOI:** 10.7759/cureus.34331

**Published:** 2023-01-29

**Authors:** Yaiseli Figueredo, Claudia Cottone, Tanira Ferreira, Jesus Gonzalez, Gianluca Iacobellis

**Affiliations:** 1 Department of Pharmacy, University of Miami Hospital, Miami, USA; 2 Department of Diabetes and Endocrinology, University of Miami Hospital, Miami, USA; 3 Department of Pulmonary Medicine, University of Miami Hospital, Miami, USA; 4 Department of Internal Medicine, University of Miami Hospital, Miami, USA; 5 Department of Medicine, University of Miami Hospital, Miami, USA

**Keywords:** extreme obesity, weight disorders, nutrition and metabolism, weight loss, glp-1 receptor agonist

## Abstract

Obesity is considered an independent risk factor for increased hospital length of stay and can be an obstacle to a safe discharge. Although typically prescribed in the outpatient setting, initiating glucagon-like peptide-one receptor agonists (GLP-1RAs) in the inpatient setting can be efficacious in reducing weight and increasing functional status.

We report the use of GLP-1RA therapy with liraglutide and subsequent transition to subcutaneous semaglutide weekly in a 37-year-old female with severe obesity, weighing 694 lbs (314 kg) with a body mass index (BMI) of 108 kg/m^2^. Multiple medical and socioeconomic factors impaired the patient from being safely discharged and ultimately led to prolonged hospitalization. The patient received 31 consecutive weeks of GLP-1RA therapy in the inpatient setting along with a very low-calorie diet (800 kcal/day). Initiation and up-titration doses were completed using liraglutide for a total of five weeks. Subsequently, the patient was transitioned to receive weekly semaglutide and completed 26 weeks of therapy. At the end of week 31, the patient’s weight decreased by 174 lbs (79 kg), or 25% of baseline weight, and BMI decreased from 108 to 81 kg/m^2^.

GLP-1RAs offer a promising avenue for weight loss interventions in patients with severe obesity in addition to lifestyle modifications. The weight loss observed in our patient at the halfway point of the total treatment duration is a milestone in the pathway to gaining functional independence and meeting the criteria for future bariatric surgery. Semaglutide, a GLP-1RA, can be an effective intervention for severely obese patients with BMI greater than 100 kg/m^2^.

## Introduction

According to the National Health and Nutrition Examination Survey (NHANES), the prevalence of obesity in the United States is about 42%, and it is estimated that about one in eleven adults have severe obesity [1]. Obesity is a preventable and treatable condition that has been associated with diabetes mellitus, cardiovascular disease, increased risk of certain cancers, and increased all-cause mortality [2]. Obesity is considered an independent risk factor for increased hospital length of stay in multiple inpatient subgroups [3]. Glucagon-like peptide-one receptor analogs (GLP-1RAs) have recently gained attention in the medical community given their robust weight loss benefits.

GLP-1 receptor activation in the small intestine and in conjunction with the central nervous system (CNS) enable various mechanisms that ultimately lead to delayed gastric emptying, increased satiety, and directly influence the appetite center of the brain [4,5]. The presence of the GLP-1 receptor within the adipocyte, as recently discovered by our group, also suggests a direct effect on the adipose tissue [6-8].

Randomized controlled trials (RCTs), such as the Semaglutide Treatment Effect in People with obesity (STEP) trials, have shown the weight loss benefit of GLP-1RAs in obese patients [9-15]. In the STEP-8 trial, the semaglutide arm had an average weight loss of 15.3 kg compared to a 6.8 kg weight loss in the liraglutide arm [15]. GLP-1RAs are currently indicated and used almost exclusively in outpatient settings. Data regarding the safety and efficacy of GLP-1RAs in the inpatient setting are scarce, although their potential was recently suggested [16].

Furthermore, there is limited evidence of GLP-1RA effects on severely obese patients with a body mass index (BMI) greater than 100 kg/m^2^. It is of relevance to this case report that when evaluating baseline characteristics of subjects enrolled in all the STEP trials, BMI did not exceed 50 kg/m^2^ [9-15].

## Case presentation

RD, a 37-year-old, white, non-Hispanic female, presented with a history of prolonged hospitalization secondary to multiple contributing medical, physical, and socioeconomic factors. The initial admission diagnosis was an acute kidney injury. The patient’s admission weight was 685 lbs (310 kg), height was 5 feet 7 inches, and BMI was 107 kg/m^2^. Her medical history was significant for hypertension, deep vein thrombosis (DVT), asthma, sleep apnea, bipolar disorder, depression, substance abuse, and stroke. Surgical history was positive for cesarean section and cholecystectomy. Regarding social history, the patient was wheelchair-bound secondary to the obese state prior to admission, uninsured, and homeless. RD’s medications prior to hospital arrival included lisinopril 40 mg by mouth daily and an albuterol inhaler as needed for shortness of breath.

The patient’s hospital stay was complicated by urosepsis and hypoxic respiratory failure requiring continuous oxygen support. Given her homeless and uninsured status, case management was unable to find an acceptable long-term care facility. Hospital dietary records show 2,000 calories per day regular cardiac diet ordered during the first eight months of hospitalization, as prescribed by an inpatient dietitian. Nursing personnel records of intake analysis validated the calories consumed by the patient and her diet adherence. Of note, a visitor was found to sneak fast food and sweets into the patient’s room repeatedly. This ultimately led to the patient gaining an additional 9 lbs from baseline hospital admission weight. Once discovered, the patient’s visitor was closely supervised by hospital personnel to avoid this behavior from occurring again.

The bariatric surgery team was consulted and recommended RD to follow up as an outpatient for bariatric intervention. Given the inability to safely discharge the patient, it was determined to start GLP-1RA therapy as an inpatient with liraglutide along with caloric restriction. The patient followed a very low-calorie diet (VLCD) of 800 calories per day. VLCD was designed by a registered dietitian ensuring a balanced macronutrient intake considering her preference. Diet compliance was documented via intake analysis by nursing staff on the electronic medical record. Liraglutide was initiated at 0.6 mg subcutaneously daily, and the dose was increased weekly at 0.6 mg increments until reaching a 3 mg daily dose. At week five of liraglutide therapy, the inpatient endocrine team was consulted and recommended switching agents to semaglutide following the findings of the STEP-8 trial. Given that the monthly cost of semaglutide and liraglutide are similar, the request to change to semaglutide was approved by hospital leadership. RD was transitioned to semaglutide 2.4 mg subcutaneously weekly on week six and completed a total of 26 weeks of semaglutide 2.4 mg dose. The patient was unblinded to pharmacologic intervention with GLP-1RA therapy. Additionally, the patient underwent physical therapy and occupational therapy two times per week. The patient also received psychological assessment and support throughout her admission.

**Figure 1:**
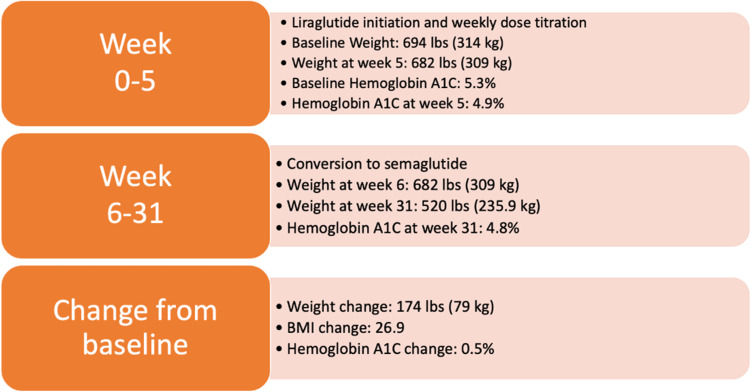
Change in weight, BMI, and hemoglobin A1C. BMI = body mass index

The patient lost a total of 174 lbs (79 kg) from the start of the GLP-1RA therapy until week 31 (Figure 1). Weight on hospital admission, 685 lbs (310 kg), was similar to weight on GLP-1RA therapy start, 694 lbs (314 kg). On hospital admission, hemoglobin A1C was 6.0%. Upon completion of 31 weeks of GLP-1 RA therapy, hemoglobin A1C was 4.8% (Figure 1). The patient tolerated the therapy well without any complaints of adverse effects. Through physical therapy and occupational therapy sessions, the patient at the end of week 31 could conduct various self-care activities such as sitting at the edge of bed and ability to dress with minimal assistance previously unable to be performed independently.

## Discussion

This case report highlights the weight loss effect potential of GLP-1RA therapy and a VLCD in a hospitalized patient with severe obesity. The studied and recommended duration of therapy with semaglutide for weight loss is 68 weeks [15]. In the STEP-8 trial, about 38% of patients in the semaglutide treatment arm were observed to have a greater than 20% weight loss after completing 68 weeks of therapy. The range of weight loss in the study was 29.5-38 lbs (13.4-17.3 kg) [15].

Findings illustrated in this case report are after 26 weeks of semaglutide therapy, or 31 weeks of GLP-1RA therapy combining the first five weeks of liraglutide, which is less than half of the recommended duration. The weight loss observed in this patient during these 31 weeks was an impressive 174 lbs (79 kg) equivalent to 25% weight loss from baseline. It is important to highlight that the weight on hospital admission, 685 lbs (310 kg), was similar to the weight on GLP-1RA therapy start, 694 lbs (314 kg). The patient in fact had a minor weight increase of 9 lbs during the first eight months of hospitalization. The weight loss observed was after the initiation of GLP-1RA and VLCD. GLP-1RA therapy was well tolerated by the patient and no adverse effects were observed. The patient was unblinded to pharmacological intervention with GLP-1RA therapy.

While GLP-1RAs are widely used and effective in the outpatient setting, there are no current guidelines for hospitalized patients. However, a recent RCT showed the beneficial effects of liraglutide versus glargine insulin at hospital discharge [17].

There is a possible preoperative role of GLP-1RA in nutrition and weight optimization prior to bariatric surgery in extreme obesity states. The maximum weight allowed for a patient to meet the criteria for bariatric surgery is 450 lbs, which is the maximum weight hospital radiology equipment can accommodate. In the postoperative phase, patients may lose as much as 60% of excess weight six months after surgery, and 77% of excess weight as early as 12 months after bariatric surgery [18,19]. A multimodal intervention combining pharmacological treatment with GLP-1RA (preoperative and postoperative), lifestyle modification, and bariatric surgery is the most effective strategy to consider for patients experiencing severe obesity with extreme BMI.

Due to the challenging conditions of this inpatient, this case report has some limitations. A baseline and week 31 dual-energy X-ray absorptiometry (DEXA) scan could not be performed. Considering the weight limitations of the equipment utilized in the hospital of 450 lbs, it was not feasible to conduct a DEXA scan. We consider this to be a limitation in our case report as it is valuable to assess body composition changes in the setting of significant weight loss, but due to the extreme body weight of the patient, it was not possible to perform a DEXA scan. A DEXA scan prior to initiating therapy and upon completion would enable precise lean and fat mass body composition changes to assess the effects of GLP-1RA on these [20]. Another limitation of this case report is the fact that it does not provide the results of a full 68-week course of semaglutide therapy.

However, it is interesting to highlight the rapid and substantial weight loss observed in about half of the anticipated duration of therapy. The inpatient multidisciplinary approach that included the endocrinologist, registered dietitian, physical/occupational therapist, and psychologist was instrumental to maximize the weight loss effect of the GLP-1RA treatment. An unplanned virtue in favor was the fact that the patient remained in a controlled hospital setting during the 31 weeks she received GLP-1RA therapy with VLCD. The inpatient setting allowed for strict control of intake and led to patient compliance with an 800-calorie diet per day without deviations. Controlling patients’ dietary intake has its challenges when conducting weight loss studies, and adherence is difficult to validate with certainty as often patient recall and history are the methods used to perform an intake analysis. This case’s unique presentation and impressive outcome highlight the value of an inpatient intervention with GLP-1RA.

## Conclusions

Data on the effect of GLP-1RA are limited in the severely obese population with extreme BMI. The weight loss benefit observed in this case report during less than half the expected treatment duration with GLP-1RA therapy was substantial and comparable to results seen upon completion of therapy in RCTs. A multimodal intervention combining pharmacological treatment with GLP-1RA, lifestyle modification, and, lastly, bariatric surgery may be the most effective strategy to consider for patients with severe obesity and extreme BMI. This case report highlights the value of using a multidisciplinary team approach in the inpatient setting to achieve maximum weight loss using GLP-1RAs.
